# Preface

**DOI:** 10.1155/S1070360899000168

**Published:** 1999

**Authors:** Harubumi Kato


					Diagnostic and Therapeutic Endoscopy, Vol. 5, p. vii
Reprints available directly from the publisher
Photocopying permitted by license only
(C) 1999 OPA (Overseas Publishers Association) N.V.
Published by license under
the Harwood Academic Publishers imprint,
part of The Gordon and Breach Publishing Group.
Printed in Malaysia.
Preface
Technological advance in endoscopic diagnosis and therapy have resulted in remarkable progress in
medical engineering. This issue examines photodynamic therapy (PDT) as a special topic. PDT differs
significantly from conventional cancer therapeutic modalities. The concept of PDT was initially reported
by Jesionek and Tappeiner in 1903. Further research on PDT was made by Dougherty who used laser
light and hematoporphyrin derivative in the 1970's, and clinical trials began in 1980. The number of
patients treated by PDT throughout the world is estimated to be more than 5,000. PDT is indicated not
only in malignant diseases but also non-malignant diseases. PDT can be used for cancer of the lung,
esophagus, stomach, colon, bile duct, skin, cervix, genital organs, urinary bladder, brain, breast and eye.
As malignant diseases tend to develop in older persons and also have a tendency toward multiple
incidence, conventional therapies are not always indicated for these patients. PDT is probably applicable
in some of those patients.
Since PDT does not damage organ function, and is a minimally invasive procedure, it has a good level
of toleration and allows rapid recovery. PDT yields a high rate of complete response in early stage cancer
and can eliminate the necessity for surgery in certain patients.
Since recent studies on photodynamic therapy were started only 30 years ago, there are still a number
of unsolved problems. However it is clear that PDT has become firmly established as one of the new
therapeutic strategies for cancer.
This issue provides an overview of the clinical experiences with PDT for cancers ofvarious organs and
attempts to define the indications and limitations of modality.
Harubumi Kato, M.D., Ph.D.
Editor-in-Chief
vii

				

